# Evidence-Based Leadership Council – A National Collaborative

**DOI:** 10.3389/fpubh.2014.00136

**Published:** 2015-04-27

**Authors:** Margaret Haynes, Susan Hughes, Kate Lorig, June Simmons, Susan J. Snyder, Lesley Steinman, Nancy Wilson, Roseanne DiStefano, Jennifer Raymond, Stephanie FallCreek, Martha B. Pelaez, Don Smith

**Affiliations:** ^1^Elder Care Services, Partnership for Healthy Aging, MaineHealth, Portland, ME, USA; ^2^University of Illinois at Chicago School of Public Health, Chicago, IL, USA; ^3^Stanford University School of Medicine, Palo Alto, CA, USA; ^4^Partners in Care Foundation, San Fernando, CA, USA; ^5^Project Enhance, Senior Services, Seattle, WA, USA; ^6^University of Washington Health Promotion Research Center, Seattle, WA, USA; ^7^Baylor College of Medicine, Houston, TX, USA; ^8^Elder Services of the Merrimack Valley, Lawrence, MA, USA; ^9^Healthy Living Center of Excellence, Boston, MA, USA; ^10^Fairhill Partners, Cleveland, OH, USA; ^11^Healthy Aging Regional Collaborative, Health Foundation of South Florida, Miami, FL, USA; ^12^Area Agency on Aging of Tarrant County, Fort Worth, TX, USA

**Keywords:** evidence-based programs, health promotion, aging, self-management

Over many years, a number of academic/community partnerships have worked independently to develop, evaluate, and bring to scale participant-centered, evidence-based self-management, and health promotion programs offered in community settings for older Americans. Many of the programs developed by these partnerships have since become critical pieces of the infrastructure that supports older adults with chronic health conditions. Indeed, community-based self-management support is an integral component of the Chronic Care Model (1) illustrated in Figure 1. This model presents elements that can improve health outcomes for people with chronic conditions, highlighting the need for connections between healthcare and community resources, integrating patient-centered, evidence-based services that empower patients. And while these programs have succeeded in finding their place in this system working independently so far, the growth and maturation of the programs, combined with the changing environment of healthcare, have prompted new collaboration among the organizations that manage and disseminate these programs, specifically, the creation of the Evidence-Based Leadership Council (EBLC).

**Figure 1:**
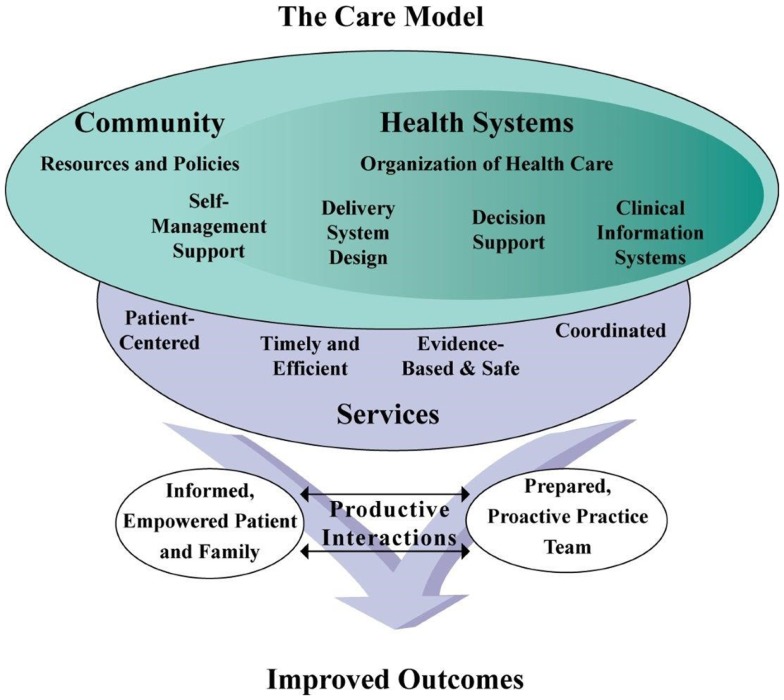
**The chronic care model**.

The EBLC is currently a group of 11 individuals representing a total of 19 evidence-based programs (Chronic Disease Self-Management suite of Programs, Matter of Balance, Enhance Fitness, Enhance Wellness, Healthy IDEAS, PEARLS, Fit & Strong!, HomeMeds, Healthy MOVES) as well as four leaders from organizations providing multiple evidence-based programs (Health Foundation of South Florida, Tarrant County Area Agency on Aging, Elder Services of the Merrimack Valley/Hebrew Senior Life, Fairhill Partners). EBLC members are employed by community-based organizations, foundations, healthcare systems, universities, and governmental entities and have been directly involved for many years in the development, evaluation, and scaling of their individual programs as well as implementation through community-based organizations. The individual program developers met informally for several years and in 2012 formed the EBLC. Over the past year, community-based organization leaders responsible for implementing multiple evidence-based programs were asked to join and be part of the council.

All the programs represented by EBLC program developers meet the Administration for Community Living’s (ACL) criteria for highest level of evidence (2). In addition to the ACL, the Centers for Disease Control and Prevention (CDC) Arthritis Program (3), Substance Abuse and Mental Health Services Administration’s (SAMHSA) National Registry of Evidence-Based Programs (4), and the Agency for Healthcare Research and Quality Innovations Exchange (5) recommend these programs and find them to be the strongest of evidence-based programs (6–14). The programs represented by the EBLC are utilized by more than 1,700 agencies in the United States with nearly 400 agencies using more than one program.

Together, the council represents more than 200 combined years of experience in developing, evaluating, scaling, implementing, and sustaining evidence-based self-management programs. All of the programs have proven effectiveness in published randomized controlled trial research and all programs have been brought to scale. The mission of the EBLC is to increase delivery of evidence-based programs that improve the health and well-being of diverse populations. The EBLC is committed to the following values:

- Person Centeredness – individuals are actively involved in programs and making a difference.
- Effectiveness – evidence-based programs focus on outcomes/results.
- Collaboration – multi-sector, multi-organizational and interdisciplinary (belief that health is achieved in the community, close to home and through broad-based collaborations).
- Equity and access – social justice, respect of diversity.
- Sustainability.

The EBLC has accomplished several important tasks, including: (1) performed an initial mapping of all agencies (more than 1,700) offering any of the 19 programs as well as which programs are being offered by each agency; (2) completed a telephone survey of 15 of the agencies offering two or more programs to identify facilitators and barriers to implementation of multiple EBPs and approaches to support scaling up these programs; (3) participated in federal meetings with the ACL, the National Council on Aging Self-Management Alliance and others; and (4) held four in-person strategic planning meetings as well as smaller subcommittee meetings and bi-weekly phone calls.

The EBLC believes that our community care system has now reached a stage where the *status quo* is no longer acceptable. As the demand for programs has increased, program infrastructures have, for the most part, not grown to meet the new demands. Each program developer has experienced challenges to keep up with increasing expectations for planning, training, and technical support, while working within the confines of their parent organization and maintaining affordability for community-based organizations. Community-based organizations have had their own set of challenges in sustaining these programs. To bring the true promise of these programs to scale, there needs to be an integration of infrastructures, and a one-stop-shop to build and to assist implementing organizations.

The focus of the EBLC going forward will be to improve coordination and efficiency around marketing, technical assistance (including readiness assessment, fidelity, implementation planning, and evaluation), training, and licensing and fee structures. An EBLC website is also being developed to improve access to tools and information in each of these areas. A shared data management platform is being expanded to include all programs in the EBLC. This platform will:

- Facilitate the effort to identify a minimal set of common data points for all programs, which can be used to evaluate dissemination, reach and outcomes both within and across programs,
- Efficiently, feed a public-facing website by providing unduplicated and jointly maintained data on organizations providing EBP trainings and workshops.
- Offer the potential for significant gains in efficiency for program owners, who can combine information about organizations providing or interested in providing EBPs and can eliminate duplication of communications and common workflow processes (e.g., licensing, training registration).
- Offer the potential for significant gains in user-friendliness for organizations providing or interested in providing EBPs, by providing a single gateway to the programs (including the common website) through which adoption research, readiness assessment, licensing, and training can be handled for one or multiple programs at a time.

The EBLC’s vision for the future is an ever increasing number of adults engaged in evidence-based programs that inform, activate, and empower them to improve their health and maintain independence. These programs will be embedded in a permanent, sustainable infrastructure –a national network supported by the EBLC’s technical assistance in implementation and dissemination, training, marketing, licensing, and evaluation. Bringing years of experience and expertise in disseminating participant-centered, evidence-based self-management, and health promotion programs in communities nationwide, the EBLC is poised to help many more organizations with limited resources effectively address population health challenges.

## Conflict of Interest Statement

The authors declare that the research was conducted in the absence of any commercial or financial relationships that could be construed as a potential conflict of interest.

This paper is included in the Research Topic, “Evidence-Based Programming for Older Adults.” This Research Topic received partial funding from multiple government and private organizations/agencies; however, the views, findings, and conclusions in these articles are those of the authors and do not necessarily represent the official position of these organizations/agencies. All papers published in the Research Topic received peer review from members of the Frontiers in Public Health (Public Health Education and Promotion section) panel of Review Editors. Because this Research Topic represents work closely associated with a nationwide evidence-based movement in the US, many of the authors and/or Review Editors may have worked together previously in some fashion. Review Editors were purposively selected based on their expertise with evaluation and/or evidence-based programming for older adults. Review Editors were independent of named authors on any given article published in this volume.
